# Enhancing Crystallization and Toughness of Wood Flour/Polypropylene Composites via Matrix Crystalline Modification: A Comparative Study of Two β-Nucleating Agents

**DOI:** 10.3390/polym14173561

**Published:** 2022-08-29

**Authors:** Shupin Luo, Chao Lv, Liang Chang, Wenjing Guo

**Affiliations:** Research Institute of Wood Industry, Chinese Academy of Forestry, No 2 Dongxiaofu, Haidian District, Beijing 100091, China

**Keywords:** wood filler, polypropylene, composite, nucleating agent, β-nucleation, crystallization behavior, mechanical properties

## Abstract

Incorporation of short wood fillers such as wood flour (WF) into polypropylene (PP) often results in a marked reduction of toughness, which is one of the main shortcomings for WF/PP composites. This research reports a facile approach to achieve toughening of WF/PP composites via introducing self-assembling β-nucleating agents into PP matrix. The effect of two kinds of nucleating agents, an aryl amide derivative (TMB5) and a rare earth complex (WBG II), at varying concentrations on the crystallization and mechanical properties of WF/PP composites was comparatively investigated. The results showed that both nucleating agents were highly effective in inducing β-crystal for WF/PP, with β-crystal content (k_β_) value reaching 0.8 at 0.05 wt% nucleating agent concentration. The incorporation of TMB or WBG significantly decreased the spherulite size, increased the crystallization temperature and accelerated the crystallization process of WF/PP. As a result of PP crystalline modification, the toughness of composites was significantly improved. Through introducing 0.3 wt% TMB or WBG, the notched impact strength and strain at break of WF/PP increased by approximately 28% and 40%, respectively. Comparatively, although WF/PP-WBG had slightly higher K_β_ value than WF/PP-TMB at the same concentration, WF/PP/TMB exhibited more uniform crystalline morphology with smaller spherulites. Furthermore, the tensile strength and modulus of WF/PP-TMB were higher than WF/PP-WBG. This matrix crystalline modification strategy provides a promising route to prepare wood filler/thermoplastic composites with improved toughness and accelerated crystallization.

## 1. Introduction

Wood plastic composites (WPC) are a series of composite materials compounding natural organic fillers (wood or non-wood) with polymer matrix, generally thermoplastic [1]. They have been burgeoning internationally in the past decades, with a variety of applications in building and construction products, automotive components, packaging and consumer goods [2,3]. Wood flour and fibers are the most widely known and used natural organic fillers in WPC, acting as filler or reinforcing material in the composite [4]. As compared to traditional inorganic fillers (e.g., talc, calcium carbonate, glass fiber and ceramics), wood fillers have advantages of low cost, biodegradability, renewability, sustainability, abundant availability, and light weight [5]. Among all the wood-source fillers, wood flour (WF), an abundant waste obtained from wood exploitation and processing residues, is widely investigated as a natural filler for thermoplastic polymers to manufacture WPC [5,6].

However, the direct incorporation of wood fillers in WPC usually leads to a marked reduction of toughness compared to neat thermoplastics, especially for wood fillers with a low aspect ratio, which is one of the main shortcomings for wood filler/thermoplastic composites [7]. Pérez et al. [8] found that the tensile strain at break of polypropylene (PP) decreased from 6.66% to 2.68% by adding 10 wt% WF. Similarly, Murayama et al. [9] found that the strain at break and impact strength of the WF/PP composites (25 wt% WF) decreased by more than 65% compared to that of PP. Sohn et al. [10] mixed varying contents of WF with PP through extrusion compounding and injection molding to prepare WPC specimens. Results showed that the impact strength decreased from 45.5 J/m to 24.7 J/m with WF contents increasing from 10 wt% to 50 wt%. The probable reasons for the decreased mechanical properties include the weak interface between wood fillers and plastic matrix, the aggregation of the wood fillers and reduced dispersibility. These are similar effects as in general for polymer-filler systems even beyond wood-based fillers [11]. At present, there are mainly two ways to improve the properties of WPC. One way is modification of wood fillers via chemical or physical treatments, such as alkali treatment [10], esterification [12], amino acid treatment [13] and heat treatment [14]. However, these treatments have evident disadvantages including the use of toxic compounds, complex processing steps and significant increase in manufacturing costs. The other common way involves addition of coupling agents such as maleic anhydride grafted polyolefins, which could promote the adhesion between polymer matrix and fillers by forming polar group interaction [9,15]. Generally, these adhesion promoters can significantly improve tensile properties of the composites, while impact strength may not be enhanced in some cases [16].

Apart from the filler and interface, the characteristics of a thermoplastic matrix such as crystalline structure and morphology are critical to the final performance of the resulting WPC as well [17,18]. The primary polymer matrices used in commercial WPCs are polyolefins, such as PE and PP, due to attractive mechanical properties, cost effectiveness, and ease of processing [2]. PP is one of the most widely used commercial thermoplastics owing to its relatively high strength and low cost [19]. PP is a typical polymorphic polymer with five different morphological forms, α, β, γ, δ and quasi-hexagonal, in which the most common and thermodynamically stable crystal form is the monoclinic α-form (α-PP) [20]. The hexagonal β-form (β-PP) is metastable but exhibits better toughness and a wider range of thermal tolerance than other forms. β-PP can be obtained through adding β-nucleating agents, which is the most effective method to generate a high ratio of β-form crystal in PP [21]. The nucleating agent acts as local nucleus in the polymer melt, which affects the nucleation, crystallization and final crystal morphology of the polymer [22].

The addition of β-nucleating agents into PP could increase nucleus density and largely induce the generation of β-crystal [23,24]. The crystallization rate and the onset crystallization temperature can be increased as well, which is beneficial for reducing cycle times in polymer processing such as extrusion or molding [25,26]. Moreover, some β-nucleating agents exhibited supramolecular self-assembly behavior upon cooling. For example, an aryl amide based nucleating agent TMB5 was reported to gradually dissolve in polymer melt at high temperature, followed by self-assembly into high aspect ratio fibrils [21,27]. Some researchers investigated the self-assembly mechanism and attributed this phenomenon to intermolecular hydrogen-bonds [28,29].

In general, most researches on WPC focus on the modification of wood filler or the improvement of the interfacial adhesion between wood filler and matrix via adding coupling agents, but few of them pay attention to the characteristics of the matrix such as crystalline structure and morphology. Although β-nucleating agents modified PP has been extensively investigated, the influence of β-nucleated modification of PP on WPC receives little attention. On one hand, in wood/PP composites wood fillers are considered as natural nucleating agents enhancing heterogeneous nucleation. One the other hand, wood fillers possess rich hydroxyl groups on the surface, so they have the potential to anchor the self-assembling nucleating agent molecules via hydrogen bonding. Therefore, the crystallization behavior of the PP matrix in the presence of both wood filler and nucleating agent remains to be discovered. The main aim of this study to investigate the effect of polymer matrix modification via adding β-nucleating agent on the crystallization and mechanical properties (especially toughness) of WF/PP composites. Two commercial β-nucleating agents with self-assembling features, an aryl amide derivative (TMB5) and a rare earth compound (WBG II), were respectively introduced into PP and then the β-nucleated PP was melt compounded with WF. The polymorphic composition, crystalline morphology, crystallization and melting behavior, and mechanical properties of the WF/PP nucleated by these two β-nucleating agents with varying concentrations were systematically compared.

## 2. Materials and Methods

### 2.1. Materials

**Figure 1:**
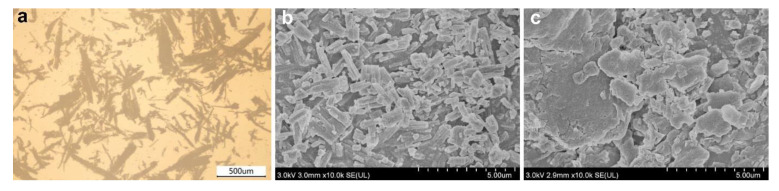
Optical micrographs of (**a**) wood flour used and SEM micrographs of (**b**) TMB5 and (**c**) WBG II.

Poplar (*Populus tomentosa* Carr.) WF of 60~100 mesh (Figure 1a) was provided by Yongdeshun mineral processing factory (Hebei, China). According to particle size analysis, the length of WF ranged between 0.8~5 mm, and its width ranged between 0.05~0.3 mm. The WF was dried at 103 °C for 24 h prior to use. Isotactic polypropylene (trade name: PP-T300) with melt flow rate (MFR) of 3 g/10 min (230 °C, 2.16 kg) was purchased from Dushanzi Petrochemical Co. Ltd. (Xinjiang, China). Two types of commercial β-nucleating agents (Figure 1b,c) were used in this research. An aryl amide derivative, N,N′-dicyclohexylterephthalamide (TMB5), was used as one β-nucleating agent. It was kindly supplied by Shanxi Provincial Institute of Chemical Industry (Shanxi, China). The rare earth compound WBG II, purchased from Guangdong Wei Linna Functional Materials Co. Ltd. (Guangdong, China), was used as another β-nucleating agent. It is a dimetal complex of lanthanum and calcium with dicarboxylic acid and amide-type ligands [29].

### 2.2. Preparation of β-Nucleated PP

Each nucleating agent (TMB5 or WBG II) and PP was thoroughly premixed in a high-speed mixer at room temperature. The rotating speed was 500 rpm and the mixing time was 5 min. Subsequently, the mixture was fed into a co-rotating two-screw extruder with the screw speed of 20 rpm. The temperature profile was 60 °C, 160 °C, 180 °C and 160 °C from the hopper to the die. The extruded long strand was air cooled and cut into approximately 4 mm granules. The obtained β-nucleated PP samples containing varying concentrations (0.05, 0.15, 0.3 and 0.5 wt%) of TMB5 or WBG II were denoted as PP-TMB_x_ or PP-WBG_x_, where subscript x represents the concentration (wt%) of nucleating agent in PP, respectively.

### 2.3. Preparation of WF/β-Nucleated PP Composites

The nucleated PP granules and WF were mixed, extruded and pelletized again under the same procedure for preparation of nucleated PP samples. The mass ratio of WF and PP was 1:9. Subsequently, the WF/β-nucleated PP granules were compression molded at 190 °C under a pressure of 5 MPa for 6 min. Then the samples were quickly moved into another press at room temperature to cool down under 8 MPa for 6 min. For comparison purposes, the specimens of pure PP and β-nucleated PP were also prepared under the same compression procedure.

### 2.4. Wide Angle X-ray Diffraction

The crystalline structure of the samples was investigated using a Bruker D8 diffractometer (operating tube voltage of 40 kV, tube current at 40 A, CuKα λ = 0.154 mm). The scanning 2θ range was 10~40°, at a scanning rate of 4°/min and a scanning step of 0.04°.

The relative content of β-form crystal (*K_β_*) present in the sample was calculated by the Turner–Jones equation [30] as follows:

(1) $$K_{\beta }=\frac{I_{\beta \left(300\right)}}{I_{\alpha \left(110\right)}+I_{\alpha \left(040\right)}+I_{\alpha \left(130\right)}+I_{\beta \left(300\right)}}$$

where *I_β(300)_* is the integral peak intensity of the *β*(300) plane, and *I_α(_*_110*)*_, *I_(α(_*_040*) *_ and *I_α(_*_130*)*_ are the integral peak intensities of α(110), α(040) and α(130) planes, respectively.

### 2.5. Hot Stage Polarizing Microscope

Crystal growth of the samples was observed in situ using an Olympus BX53 polarizing microscope (POM) equipped with a hot stage and a CCD color camera. A thin film of the sample was sandwiched between two microscope cover slips and placed in the hot stage. Samples were first heated to 200 °C and kept for 5 min to eliminated the thermal history, and then cooled to room temperature at a rate of 5 °C/min. The morphologies of the crystal were recorded at constant time intervals during the non-isothermal crystallization process.

### 2.6. Differential Scanning Calorimetry

Thermal behaviors of pure PP, nucleated PP, and its composites were measured using a PerkinElmer DSC 8000 differential scanning calorimeter in a flowing nitrogen atmosphere (20 mL/min). For non-isothermal crystallization, the sample (about 5 mg) was heated from 30 °C to 200 °C at a rate of 30 °C/min and held isothermally for 5 min to eliminate thermal history, and then cooled to 40 °C at different cooling rates of 5, 10, 20 and 40 °C/min, respectively. Subsequently, the sample was reheated to 200 °C at a rate of 10 °C/min.

### 2.7. Mechanical Tests

The izod notched impact strength was tested using a Zwick/Roell HIT50P pendulum impact tester according to ISO 180. The specimens were rectangular bars (80 × 10 × 4 mm^3^) with a 45° V-notch of 2 mm depth. The tensile properties were measured using an Instron5582 universal testing machine according to ISO 527. The tested specimens were dumbbell shaped bars with the length of 150 mm and the thickness of 4 mm. The crosshead speed was 5 mm/min. The crosshead speed was 2 mm/min. All mechanical properties were measured under ambient temperature, and the reported values are the average of eight specimens.

## 3. Results and Discussion

### 3.1. Polymorphic Composition in the β-Nucleated PP and WF/β-Nucleated PP

**Figure 2:**
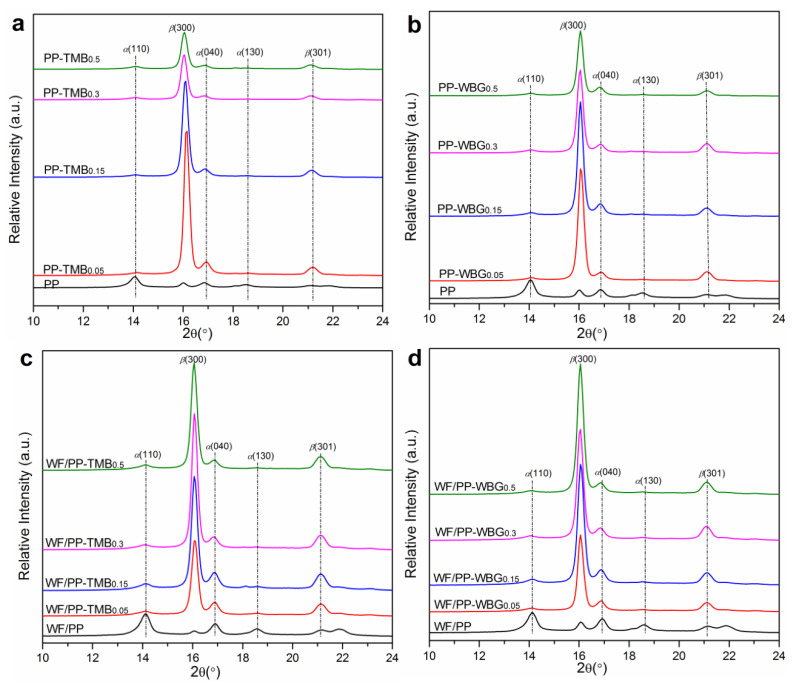
WAXD spectra of pure PP, (**a**) PP-TMB, (**b**) PP-WBG, (**c**) WF/PP-TMB, and (**d**) WF/PP-WBG containing varying concentrations of TMB5 or WBG II.

Wide angle X-ray diffraction (WAXD) was performed to characterize the polymorphic composition in the compression molded samples. As shown in Figure 2, pure PP exhibited three typical diffraction peaks at 2θ = 14.0°, 16.8° and 18.5°, corresponding to the (110), (040), (130) planes of the α-form crystal, respectively. For PP-TMB and PP-WBG samples, the characteristic peaks of α-form crystal at 2θ = 14.0° and 18.5° were extremely weak. Meanwhile, noticeable characteristic peaks at 2θ = 16.1° corresponding to the (300) plane of β-form crystal appeared. This indicates that TMB5 and WBG II largely induced the formation of β-PP crystals. The WAXD spectrum of WF/PP was similar to that of PP, indicating that the presence of WF did not change the crystal form of PP. For WF/PP-TMB and WF/PP-WBG composites, the characteristic peaks of β(300) and β(301) planes were also observed, suggesting the formation of β-PP crystals in the WF filled composites.

**Figure 3:**
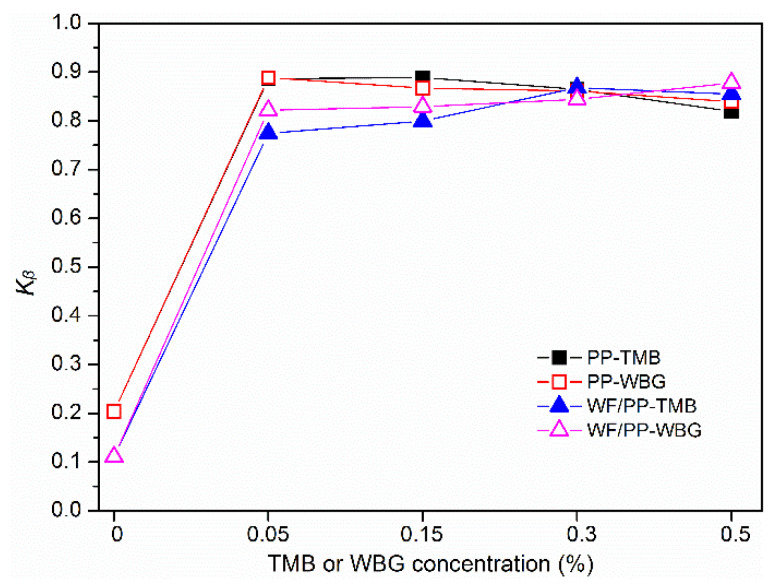
Variation of relative content of β-form crystal (*K_β_*) with TMB5 or WBG II concentration for PP-TMB, PP-WBG, WF/PP-TMB, and WF/PP-WBG.

The relative content of β-crystal (*K_β_*) was calculated and the results are presented in Figure 3. For PP-TMB and PP-WBG samples, the addition of 0.05% TMB or WBG sharply increased the *K_β_* value to approximately 0.89, verifying the high efficiency of these two kinds of nucleating agents. After that, the *K_β_* value showed no substantial increase when nucleating agent concentration further increased. Similarly, previous studies on β-nucleated PP reported critical nucleating agent concentrations of 0.05–1% above which the *K_β_* leveled off [25,31].

For WF/PP-TMB composites, the *K_β_* first increased rapidly with the addition of 0.05% TMB5, and then increased slowly to the maximum value of 0.87 at 0.3% TMB5 concentration. For WF/PP-WBG composites, the *K_β_* also first increased sharply with adding 0.05% WBG II. It then increased at a lower rate with the WBG concentration up to 0.5%. Generally, WF/PP-WBG composite had a slightly higher *K_β_* value than WF/PP-TMB composite at the same concentration.

### 3.2. Crystalline Morphology

**Figure 4:**
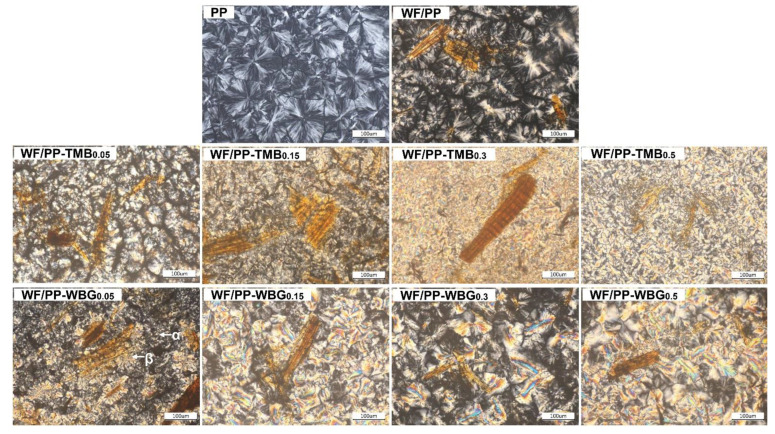
Polarized optical micrographs of pure PP, WF/PP, WF/PP/TMB, and WF/PP/WBG containing varying concentrations of TMB5 or WBG II after non-isothermal crystallization at cooling rate of 5 °C/min.

The morphology development of spherulites in pure PP and WF/PP containing varying contents of TMB5 or WBG II during cooling was observed by POM. Figure 4 shows the optical micrographs of final crystalline morphology. Pure PP exhibited only isotropic α-form spherulites, and there are clear boundaries between the spherulites. Incorporation of WF induced heterogeneous nucleation of PP. It is observed that some spherulites formed on the surface of WF in WF/PP. With the addition of TMB5 or WBG II, the morphology of PP altered dramatically.

For WF/PP-TMB composites, as the TMB5 concentration was 0.05%, smaller β-PP spherulites with strong contrast were formed, revealing the strong β-nucleating effect of TMB5 on the crystallization of WF/PP composites. When the TMB5 concentration further increased to 0.15%, PP matrix dominantly crystallized in the form of β-crystal and the boundary between spherulites became blurred. For WF/PP-TMB_0.3_, more concentrated β-crystals with highly birefringent character were generated. For WF/PP-TMB_0.5_, bright anisotropic β-crystals were largely induced. This phenomenon was attributed to TMB5 self-assembling into the form of needle structure in PP melt, on which the bright β spherulites grew in the lateral direction during the crystallization process. Similar phenomenon has been observed by several previous studies on TMB5 nucleated PP [25,27].

For WF/PP-WBG composites, the co-existence of α-spherulite and bunching β-spherulites was clearly observed at 0.05% WBG concentration. Furthermore, the size of spherulites in WF/PP/ WBG_0.05_ was much smaller than that in pure PP. Meanwhile, β-spherulite agglomerates developed on the surface of WF, forming a transcrystalline structure. When the WBG II concentration increased to 0.15%, the size of β-crystals increased due to aggregation of supermolecular structures of β-crystal, which shows strong birefringence. It is noted that the agglomerate state of supermolecular structure changed with varying WBG concentrations. For WF/PP-WBG_0.3_, β-crystal of strip-like structure was clearly observed. For WF/PP-WBG_0.5_, flower-like β-crystals were formed without obvious boundary. Several research groups observed similar crystalline morphology evolution in PP containing various WBG concentrations [23,31,32]. They demonstrated that the final crystalline structure depended largely on the self-assembly structure of the nucleating agent during cooling.

In comparison to WF/PP/WBG composites, the amount of β-crystal in WF/PP/TMB was higher and its size was much smaller. The different crystalline morphology of WF/PP-TMB and WF/PP-WBG composites was likely related to the microstructure of these two nucleating agents. As shown in Figure 1, TMB5 is composed of cubic granules with nanometer-scale length, while WBG II is composed of irregular flakes with wide size distributions.

### 3.3. Non-Isothermal Crystallization and Melting Behaviors

**Figure 5:**
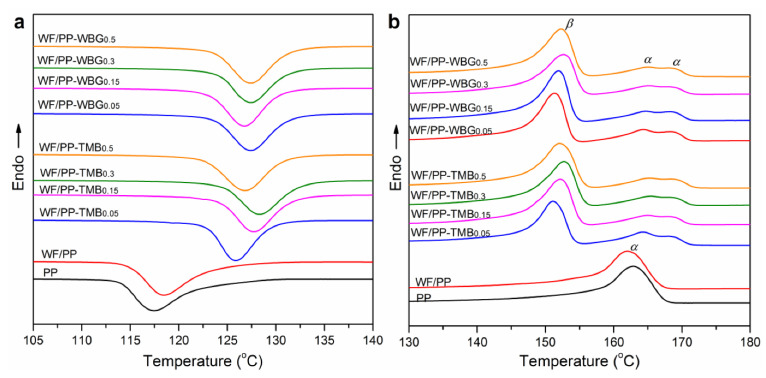
DSC (**a**) crystallization and (**b**) subsequent melting thermograms of pure PP, WF/PP, WF/PP-TMB and WF/PP-WBG containing varying concentrations of TMB5 or WBG II at cooling and melting rate of 10 °C/min.

DSC analysis was adopted to investigate the non-isothermal crystallization and subsequent melting behaviors of pure PP and WF/β-nucleated PP composites. Figure 5 shows the crystallization and subsequent melting thermograms at a cooling and melting rate of 10 °C/min. The crystallization peak of PP slightly shifted to higher temperature with the addition of WF, attributed to the heterogeneous nucleation effect of WF. A similar phenomenon was also observed in WF filled poly(lactic acid), in which WF provides heterogeneous nuclei and thus enhanced the nucleation density [33]. For WF/PP composites, the addition of TMB5 or WBG II produced a marked shift of the crystallization peak towards higher temperature, implying that the used nucleating agents promoted the crystallization of PP matrix during non-isothermal crystallization process. The crystallization temperature increased with increase in TMB5 or WBG II concentration below 0.3%, and it slightly decreased with TMB5 concentration increasing to 0.5%, whereas it stayed constant when the WBG concentration increased to 0.5%.

For subsequent melting traces as shown in Figure 5b, pure PP and WF/PP exhibited only one endothermic melting peak around 162 °C, which was associated with the melting of α-crystal. TMB or WBG modified WF/PP exhibited three melting peaks of about 152 °C, 165 °C and 169 °C, corresponding to the melting of the β-form, original α-form and more perfect α-form derived from recrystallization, respectively [31]. The melting peak area of α-crystal significantly decreased with the incorporation of TMB or WBG. Moreover, the peak area of β-crystal was much bigger than that of α-crystal in WF/PP-TMB and WF/PP-WBG composites. These results verified the formation of massive β-crystals in WF/PP composites via incorporating TMB5 or WBG II. With the increase of the nucleating agent concentration, the melting peak temperature of β-crystal slightly increased up 2 °C to with 0.3% TMB or WBG, and then decreased with 0.5% concentration. It appears that when TMB or WBG were added in low concentration, PP molecules crystallized in the β-crystal with insufficient crystalline perfection. With the increase of concentration, a higher density of crystal centers was achieved, which accelerated the growth of β-crystal and promoted the crystalline order [34]. As a result, the melting point was increased. However, when excessive nucleating agent was introduced, the aggregation of nucleators occurred and its solubility in PP melt was limited, which would reduce the nucleation efficiency.

**Figure 6:**
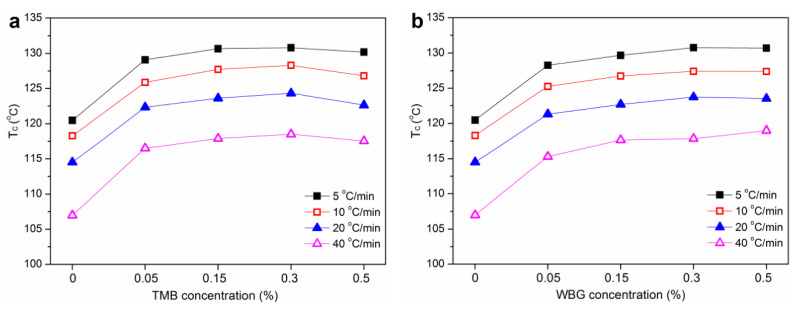
Variation of crystallization peak temperature (*T_c_*) with nucleating agent concentration and cooling rate for (**a**) WF/PP-TMB and (**b**) WF/PP-WBG.

The crystallization peak temperature (T_c_) values versus nucleating agent concentrations at different cooling rates (5, 10, 20 and 40 °C/min) for WF/PP-TMB and WF/PP-WBG are presented in Figure 6. Cooling rate had a remarkable effect on the crystallization of the samples. The T_c_ decreased with increasing cooling rate, as is typical for polymer crystallization. At higher cooling rates, less time is available to overcome the energy barriers for nucleation, so the crystallization starts at lower temperatures [25]. The T_c_ of WF/PP at a cooling rate of 10 °C/min was around 118.3 °C. Given the same cooling rate, the T_c_ increased sharply with adding 0.05% TMB or WBG, and then increased at a lower rate with further addition of nucleating agent. The maximum T_c_ for WF/PP-TMB and WF/PP-WBG samples was observed at 0.3% TMB at 0.5% WBG concentrations, respectively. At a cooling rate of 10 °C/min, the maximum T_c_ was 128.3 °C for WF/PP-TMB_0.3_ and 127.4 °C for WF/PP-WBG_0.5_. The variation trend of T_c_ was consistent with that of *K_β_*, indicating that the formation of β-crystal promoted the crystallization of WF/PP composites.

### 3.4. Non-Isothermal Crystallization Kinetics

The relative crystallinity (*X*) as a function of temperature (*T*) can be obtained by integrating the partial areas under the DSC crystallization thermogram according to the following equation [35]:

(2) $$X_{T}=\frac{X_{T}\left(T\right)}{X_{T}\left(\infty \right)}=\int_{T_{0}}^{T}\left(\frac{dH}{dT}\right)dT/\int_{T_{0}}^{T_{\infty }}\left(\frac{dH}{dT}\right)dT\;$$

where *X_T_(T)* and *X_T_(∞)* represent the absolute crystallinity at temperature *T* and at the termination of the crystallization process, respectively. *T*_0_ and *T_∞_* are the onset and offset temperature of crystallization, respectively, and *dH/dT* is the heat flow rate. For non-isothermal crystallization at different cooling rates (ø), the temperature-scale can be converted into time-scale by the following equation [36]:

(3) $$t=\left(T_{0}-T\right)/\emptyset$$

**Figure 7:**
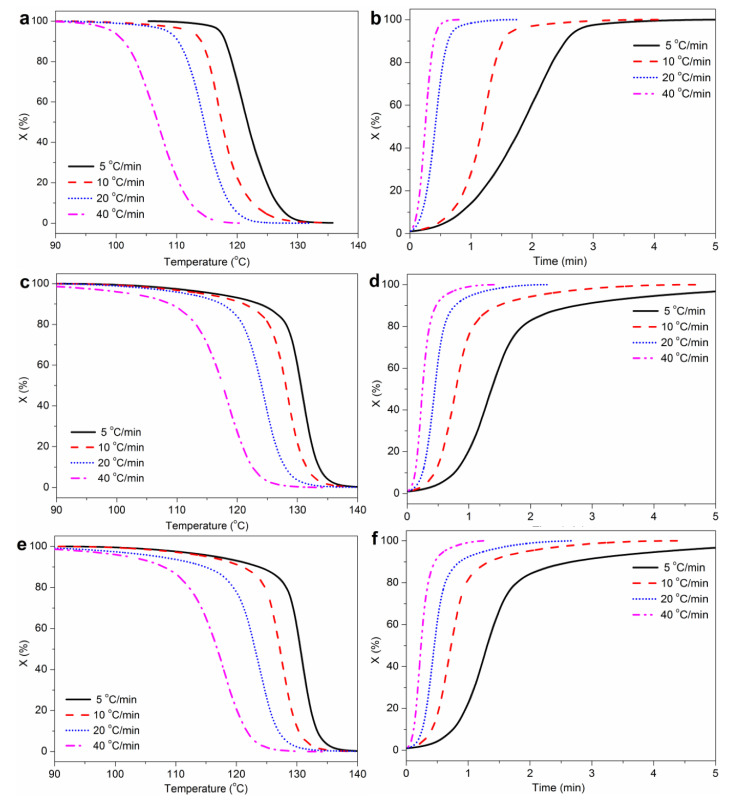
Relative crystallinity (X) as a function of temperature or time for non-isothermal crystallization of (**a**,**b**) WF/PP, (**c**,**d**) WF/PP-TMB_0.3_ and (**e**,**f**) WF/PP-WBG_0.3_.

The relative crystallinity *X* as a function of temperature for WF/PP, WF/PP-TMB_0.3_ and WF/PP-WBG_0.3_ samples at various cooling rates are showed in Figure 7a. Increasing the cooling rate resulted in the shift of crystallization process to lower temperature, and the range of crystallization temperatures broadened. This is attributed to the fact that the motion of polymer chains lagged at a higher cooling rate [36]. At the same cooling rate, the plots of *X* versus temperature for WF/PP-TMB_0.3_ and WF/PP-WBG_0.3_ shifted markedly towards the right compared to WF/PP, indicating that the crystallization occurred at higher temperatures when incorporating TMB or WBG.

Figure 7b shows the relative crystallinity *X* as a function of time at various cooling rates, which exhibits sigmoidal growth. The time for crystallization completion decreased at higher cooling rates. To quantitively evaluate the crystallization rate and ability, the half time of crystallization (t_1/2_) was determined at 50% crystallinity. Generally, the smaller the value of t_1/2_, the shorter the time needed for the crystallization process [37]. As shown in Table 1, the t_1/2_ values of all the samples were markedly decreased with increasing cooling rate. Furthermore, at the same cooling rate the t_1/2_ value for WF/PP-TMB_0.3_ and WF/PP-WBG_0.3_ was smaller than that of WF/PP, especially in the case of lower cooling rate. For instance, at the cooling rate of 10 °C/min, the t_1/2_ value of WF/PP decreased from 1.19 min to 0.80 min and 0.73 min when 0.3% TMB5 or WBG II was incorporated, proving that the incorporation of TMB or WBG accelerated the crystallization process. Besides, the t_1/2_ value of WF/PP-WBG was lower than WF/PP-TMB at the same cooling rate, suggesting the better crystallization ability of WF/PP-WBG. This result agrees with the findings from XRD analysis.

To further investigate the nucleation mechanism, the modified Avrami method was used to determine the non-isothermal crystallization kinetics parameters [35], as described by the following equations:

(4) $$ln\left[-ln\left(1-X_{t}\right)\right]=ln\;k_{t}+n\;ln\left(t\right)$$

(5) $$\ln k_{c}=\ln k_{t}/\emptyset$$

where *k* is crystallization rate constant, *n* refer to the Avrami exponent, and *k_c_* is the kinetic crystallization rate. Accordingly, by plotting *ln[−ln(1−X_t_)]* versus *lnt*, the values of *n* and *k* can be obtained from the slope and intercept by linear fit.

The detailed non-isothermal crystallization kinetic parameters for WF/PP, WF/PP-TMB_0.3_ and WF/PP-WBG_0.3_ are listed in Table 1. The value of *n* strongly depends on the nucleation and crystal growth, and *k* represents the crystallization rate [38]. Values of *n* for the three samples at all cooling rates varied between 2.49 and 3.42, which was attributed to a heterogeneous nucleation followed by three-dimensional growth of crystallites [39,40]. The value of *k_c_* increased with increasing cooling rate. At a given cooling rate, the *k_c_* of WF/PP-TMB_0.3_ and WF/PP-WBG_0.3_ was higher than that of WF/PP, indicating that the crystallization of WF/PP was promoted via the incorporation of TMB5 or WBG II.

### 3.5. Mechanical Properties

**Figure 8:**
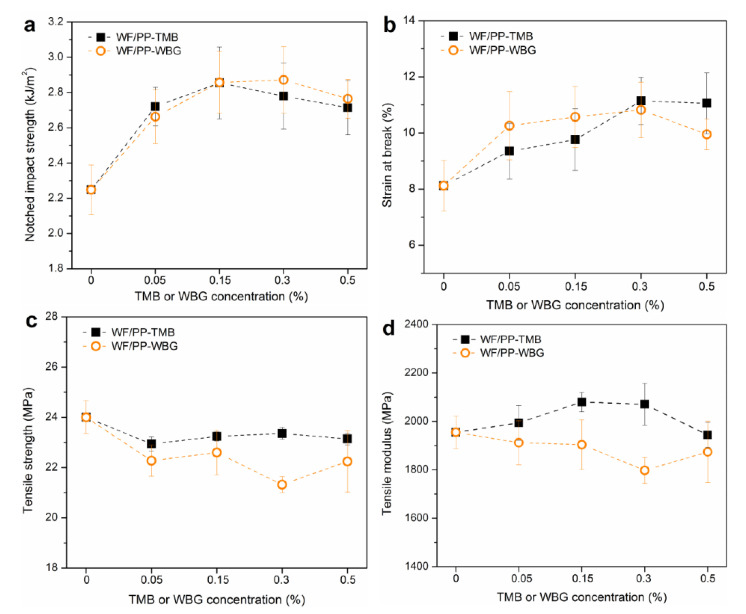
Mechanical properties of WF/PP, WF/PP-TMB and WF/PP-WBG composites: (**a**) Notched impact strength; (**b**) Strain at break; (**c**) Tensile strength; (**d**) Tensile modulus.

Impact strength is directly related to the ability of a material to absorb energy during the fracture process, which highly depends on material cohesion [17]. The izod notched impact strength is considered to reflect toughness directly [23]. WF/PP showed high discontinuity due to the finely dispersed WF particles in the PP matrix, thus resulting in low cohesion and brittle composites. As shown in Figure 8a, the impact strength of WF/PP composites was improved by incorporating 0.05% TMB or WBG. The maximum value in impact strength was observed when 0.15% TMB or 0.3% WBG was added. After that, the impact strength decreased with further increasing concentration. The impact strength of WF/PP-TMB_0.15_ and WF/PP-WBG_0.3_ increased by 27% and 28% compared to that of WF/PP, respectively. The variation trend of notched impact strength is basically consistent with that of *K_β_*, indicating that the improvement in toughness is related to the increased content of β-form crystal in the matrix.

As another indicator reflecting toughness, the strain at break also increased with the incorporation of TMB or WBG (Figure 8b). For WF/PP-TMB, the strain at break increased with TMB concentration increasing from 0.05% to 5%. For WF/PP-WBG, the strain at break increased with the WBG concentration up to 0.3% and then levelled off. When 0.3% nucleating agent was incorporated, the strain at break of WF/β-nucleated PP increased by approximately 40% compared to WF/PP. It is noted that when nucleating agent concentration exceeded 0.3%, the strain at break of WF/PP-TMB was higher than WF/PP-WBG although WF/PP-WBG had a higher content of β-crystal. This was related to the crystalline morphology, with WF/PP-TMB_0.3_ and WF/PP-TMB_0.5_ exhibiting a more uniform distribution of crystals than WF/PP-WBG_0.3_ and WF/PP-WBG_0.5_. With increase in WBG concentration, big-size β-crystal agglomerates were formed in WF/PP-WBG (Figure 4), which may cause stress concentration. Overall, incorporation of about 0.3% nucleating agent was found to toughen the WF/PP most under the processing conditions in this study.

The tensile strength of WF/β-nucleated PP was reduced compared to WF/PP (Figure 8c). For tensile modulus, TMB and WBG exhibited different effects on the composites (Figure 8d). Incorporation of TMB5 increased the tensile modulus of the resulting WF/PP, exhibiting maximum tensile modulus at 0.15% concentration. In contrast, incorporation of WBG II decreased the tensile modulus of the resulting composites. Comparably, the tensile strength and modulus of WF/PP-TMB were higher than WF/PP-WBG.

Previous research by Luo et al. [23,31] demonstrates that the mechanical properties of β-nucleated PP depend on both the β-form content and the crystalline morphology. They found that there was a critical nucleation content above which the crystalline morphology might dominate the toughness of PP instead of the β-crystal content. Similarly, two concentration ranges may be distinguished in this study: (1) as the nucleating agent concentration was 0.05%, the β-crystal content and toughness increased sharply compared to neat WF/PP; (2) when the nucleating agent concentrations were in the range of 0.15–0.5%, the content of β-crystal began to level off, while agglomerates of β-crystals were obtained due to self-assembly of nucleators. Thus, the crystalline morphology in WF/PP varied with increasing concentration. In addition, the final temperature of heating was set as 190 °C in this study, due to preventing the thermal degradation of WF. Under such processing temperatures, the solubility of the nucleating agents was limited and the nucleators were not completely dissolved in the PP melt, especially at higher concentrations, which would affect the efficiency of nucleation.

## 4. Conclusions

This study reported a practical strategy to promote toughness and crystallization of WF/PP composites via introducing β-nucleating agent into PP matrix. The effects of two commercial nucleating agents (TMB5 and WBG II) at varying concentrations (0.05%, 0.15%, 0.3% and 0.5%) on the crystallization and mechanical properties of WF/PP composites were systematically investigated. Both nucleating agents largely induced β-crystals in WF/PP composites, along with a considerable increase in T_c_ and crystallization rate. Compared to neat WF/PP, the density of spherulites in WF/β-nucleated PP composites was significantly increased and their size was reduced. Moreover, aggregation of β-spherulites around WF was observed in WF/PP-WBG composites, probably due to the hydrogen bonding between WF and WBG, as well as self-assembly behavior of WBG. The incorporation of TMB or WBG significantly increased the impact strength and the strain at break of WF/PP. In general, WF/PP-WBG composite exhibited slightly higher content of β-crystal and better toughness than WF/PP-TMB, while its tensile strength and modulus were lower than that of WF/PP-TMB. This matrix-based crystalline modification method via introducing a nucleating agent is feasible for improving the toughness of WPC, as well as accelerating the crystallization process in WPC production.

## Figures and Tables

**Table 1:** Non-isothermal crystallization kinetic parameters for WF/PP, WF/PP-TMB_0.3_ and WF/PP-WBG_0.3_.

Sample	Cooling Rate (°C/min)	n	t_1/2_ (min)	*k_c_* (min^−n^)
WF/PP	5	3.42	1.83	0.03
	10	3.38	1.19	0.23
	20	2.92	0.47	0.52
	40	2.86	0.25	0.80
WF/PP-TMB_0.3_	5	3.23	1.38	0.05
	10	3.29	0.80	0.27
	20	3.22	0.45	0.58
	40	2.64	0.25	0.83
WF/PP-WBG_0.3_	5	3.35	1.32	0.05
	10	3.36	0.73	0.27
	20	3.15	0.44	0.58
	40	2.49	0.24	0.84

## Data Availability

Not applicable.
